# We Need Strong Public Health Care to Contain the Global Corona Pandemic

**DOI:** 10.1177/0020731420916725

**Published:** 2020-03-18

**Authors:** Wim De Ceukelaire, Chiara Bodini

**Affiliations:** 1 Viva Salud, Brussels, Belgium; 2 People’s Health Movement, Brussels, Belgium

**Keywords:** COVID-19, privatization, China, corona virus

## Abstract

The corona virus (COVID-19) outbreak has spread from China to over a hundred countries in less than 2 months. Now is the time to take stock and to assess the responses of different countries to the outbreak so far. What we can learn from the global Corona pandemic so far is that strong public health systems have the resilience to address massive health threats with the collective responses they require. Privatization of health services and individualization of risks might further undermine our ability to address this and future global pandemics.

The corona virus (COVID-19) outbreak has spread from China to over a hundred countries in less than 2 months. As of March 10, confirmed cases were above 100,000 and deaths over 4,000. Now is the time to take stock and to assess the responses of different countries to the outbreak so far.

The report of the WHO–China joint mission on COVID-19 (https://www.who.int/docs/default-source/coronaviruse/who-china-joint-mission-on-covid-19-final-report.pdf) offers essential insights in containment strategies, explains why health systems in many parts of the world are much less capable of implementing them, and shows the way towards more resilient health systems.

The report concludes that China has rolled out perhaps the most ambitious, agile and aggressive disease containment effort in history. And it did this with success. Cases have decreased considerably and daily life is slowly resuming in areas that have been under huge stress in the past few weeks.

As there is no vaccine nor specific treatment, the containment of the outbreak is based on a number of measures including identifying people who are sick, bringing them to care, following up on contacts, preparing hospitals and clinics to manage a surge in patients, and training health workers (https://www.who.int/news-room/detail/07-03-2020-who-statement-on-cases-of-covid-19-surpassing-100-000). The WHO–China joint mission report’s conclusion is remarkable as it says that this has only been possible “due to the deep commitment of the Chinese people to collective action in the face of this common threat.”

Similar conclusions can be drawn from experiences in other Asian territories. In an article on the website of *The Lancet* (https://www.thelancet.com/journals/lancet/article/PIIS0140-6736(20)30551-1/fulltext), experts assess resilience of health systems in Hong Kong, Singapore, and Japan according to their ability to apply adequate containment strategies with regard to the current COVID-19 crisis. Health systems in these locations have generally been able to adapt.

The 3 locations introduced appropriate containment measures and governance structures; took steps to support health care delivery and financing; and developed and implemented plans and management structures. Integration of services in the health system and across other sectors has amplified the ability to absorb and adapt to shock. Besides, this experience has demonstrated that the trust of patients, health care professionals, and society as a whole in government is of paramount importance for meeting health crises. Interestingly, also Singapore, Japan, and Hong Kong rely on strong public health systems that enjoy wide support and that are able to reach and mobilize the population beyond health workers.

In many countries across the globe, public state-funded and government-run health systems have been gradually dismantled. Privatization has affected their ability to coordinate large-scale preventive campaigns, limited their capacity to expand curative services in crisis situations while eroding the broad public’s confidence in the health system as a whole.

For example, in order to apply effective contact tracing, a fine-grained health system with an expanded first line is imperative. China has been able to mobilize thousands of health workers in the efforts to track down the contacts of infected individuals. In the US, where primary health care is weak and the health system is highly dependent on secondary and tertiary care, large-scale contact tracing is almost impossible.

Now that COVID-19 is rapidly spreading to Europe and the US, we might witness the vulnerability of more privatized health systems. In Italy, the European country worst hit by the epidemic, the regionalization of health care – very much part of a broader design to progressively dismantle and privatize the national health care service (NHS) – has significantly delayed the adoption of coherent measures to contain the disease and strengthen the health system.

As their health systems are unable to coordinate adequate collective responses, it is not surprising that the measures taken by European governments are calling on people’s individual responsibilities. Social distancing has become the cornerstone of their COVID-19 mitigation plans.

Of course it is correct to call on people’s individual responsibility and it’s true that social distancing has also played a role in China’s containment of the virus outbreak but it’s also important to acknowledge that these measures are inadequate to handle large-scale health threats. What we can learn from the global Corona pandemic is that strong public health systems have the resilience to address massive health threats with the collective responses they require. Privatization of health services and individualization of risks might further undermine our ability to address this and future global pandemics.

